# Exploring the Joint Association of Road Traffic Noise and Air Quality with Hypertension Using QGIS

**DOI:** 10.3390/ijerph20032238

**Published:** 2023-01-27

**Authors:** Wisdom K. Adza, Andrew S. Hursthouse, Jan Miller, Daniel Boakye

**Affiliations:** 1 School of Computing, Engineering & Physical Sciences, University of the West of Scotland, Paisley PA1 2BE, UK; 2 School of Health & Life Sciences, University of the West of Scotland, Hamilton G72 0LH, UK

**Keywords:** environmental noise, air quality, environmental pollution, transportation, environmental public health, cardiovascular disease, quantum geographic information system (QGIS)

## Abstract

There is growing evidence linking exposure to air pollution and traffic noise with hypertension. The aim of this study was to examine the associations of registered hypertension cases and hypertension rate with exposure to air pollution and road noise. In this cross-sectional study, we linked the information from the NHS Scotland database of 776,579 hypertension patients’ registrations and rates per 13.80 people at the Scottish NHS Board, HSCP, Cluster, and GP practice levels. Based on the geospatial attributes, the data on residential areas were added by modelling annual average air pollutant concentrations, including particulate matter (PM_10_ and PM_2.5_), nitrogen dioxide (NO_2_), and road-traffic noise at different frequency components (Lden). The relationships between exposure to road noise, air pollution, and hypertension were examined using multiple regression and multivariate analysis. Traffic noise and air pollution at various frequency components positively and negatively predicted registered hypertension cases and hypertension rate. Based on the canonical loading technique, the variance explained by the canonical independent variable at a canonical correlation of 0.342 is 89%. There is a significant correlation between joint air pollution and noise at different frequency components and combined registered hypertension cases and hypertension rate. Exploring the combined effects of the two environmental exposures and the joint modelling of noise and air pollutants with hypertension in geospatial views provides an opportunity to integrate environmental and health data to support spatial assessment strategies in public and environmental health.

## 1. Introduction

Urbanisation, which contributes to noise and air pollution, is growing. Between 2007 and 2017 it increased by 55.4% in the UK [1].

For the UK, data from a health survey estimating the prevalence of hypertension for areas in England revealed that about 11.8 million adults had hypertension, equating to 22.6% of the population [2]. The Scottish Public Health Observatory [3] estimates that the risk of hypertension is high and can lead to significant morbidity. In 2019 the prevalence sharply increased among people aged 16 years and above [3]. Hypertension in Wales increased by 15.8% between 2018 and 2019 and is currently the most prevalent morbidity [4]. Nonetheless, according to the Northern Ireland Quality and Outcome Framework Statistics (QOF), the rate of hypertension is 14.0%, making it the most prevalent morbidity in Northern Ireland [5].

Given the urgency for hypertension prevention [6,7], understanding the environmental impact that combined noise and air concentrations have on community households is crucial [8]. Consequently, it is pertinent to study the potential impact of exposure to multiple traffic noise sources at different frequency components and air pollutant concentrations. By assessing potential risk across communities this study aims to support best clinical practice for exposed urban population,, to raise awareness of the potential significance of expected hypertension.

Information on the prevalence of hypertension is generally hard to access [9]. However, data on hypertension cases related to noise and air pollutants are being produced. The potential environmental and health challenges posed by excessive traffic emission and associated traffic-related air pollution (TRAP) are heavily influenced by liveability conditions, neighbouring environmental features, and the socioeconomic status of affected households, particularly in densely populated areas, for example seen in Hong Kong [10].

As a result, it is critical to first incorporate “social determinants of health” into the retrieval of local-scale traffic emission distributions and then study the potential links between citizens, well-being, and health quality via air quality modelling [10].

In this case study determinants include social patterns, such as working hours, usual peak hours, citizen traffic mobility, the character of the area and population density, as well as the building typologies located on various streets. Considering the “official working hours” of offices and private companies, as well as the operational timeslots of retail shops in business districts (e.g., commercial areas), it is expected that the traffic flow between most residential districts and nearby commercial districts will be the heaviest during rush hours (i.e., early morning and evening periods); thus, the highest NO_x_ and PM_2.5_ emissions should also be detected during these timeslots [10].

While pollution concentrations are lower at midnight due to lower traffic loading, the residual levels still have an impact. It still affects residents living near public roads, particularly in Hong Kong, due to tall building typologies near roadside transportation routes under the constraints of limited space for utilisation [10].

The case study also attempted to develop a systematic geo-processing framework via data analytics, which processes available hourly traffic NO_x_ and PM_2.5_ emission records of all roads in Hong Kong, to obtain a more realistic distribution of district-wise traffic emissions for the modelling of spatial pollution profiles, which may open new windows for conducting health exposure assessment, particularly during peak hours [10].

Road traffic is a crucial source of environmental noise, and studies have revealed the impact of traffic noise on cardiovascular issues, including myocardial infarction and hypertension [11,12]. The effects of exposure to traffic on the body frequently include irritation and sleep disturbance. Furthermore, epidemiological studies have found that environmental noise is associated with increased arterial hypertension, myocardial infarction, heart failure, and stroke [13,14,15,16]. Disease associated with PM_2.5_ exposure include respiratory tuberculosis and malignant neoplasms of the trachea, bronchi, and lungs. The other respiratory and cardiovascular system diseases include diabetes mellitus, ischemic heart disorders, pulmonary embolism, cerebral vascular diseases, pneumonia, acute lower respiratory infections, and chronic lower respiratory diseases [10].

Disorders, such as pulmonary tuberculosis, malignant neoplasms of the trachea, bronchus, and lung, and lower respiratory diseases, are related to NOx traffic emissions [10].

In many places, vehicle emissions are the sources of high levels of nitrous oxide (NO) and particulate matter (PM) [17]. Particularly close to large roadways, the severity and duration of traffic congestion can significantly increase pollutant emissions and worsen air quality [17]. According to a previous study, significant health hazards may be associated with traffic congestion. Increasing traffic can significantly increase the risks, depending on the road style and other variables. Danger brought on by traffic congestion must consider travel time, length of rush hour, estimates of the emissions caused by the congestion, and uncertainty. These emissions raise the morbidity and mortality risks for motorists, commuters, and those living close to roads [17].

There is growing evidence that outdoor exposure, air pollution, and traffic noise are associated with poor mental health. Most research has only examined one of these two related exposures with associated poor mental health. These studies run the danger of overstating the effect of poor mental health because of the analysed exposure while underestimating the overall impact of the combined environmental exposures [18].

Observational studies show that nighttime noise dramatically raises stress hormone and oxidative stress levels in the blood, which may cause endothelial dysfunction and arterial hypertension [19]. Regarding the influence on cardiovascular health, some cohort studies in the UK have substituted the distance to major roadways for exposure to air pollutants and noise pollution [20,21]. Other studies have also used spatial and temporal distributions to investigate road traffic noise and air pollution associations with cardiovascular outcomes [22,23]. However, a research gap exists regarding the impact of the combined association of road traffic noise at different frequencies and air pollution on hypertension outcomes [24].

In this study we address this research gap by using GIS models [25] to evaluate the relationship between combined air concentration and noise at different frequency components of pollution with hypertension. We provide new insight to the application of model development for the epidemiological assessment of hypertension related to environmental noise and air pollution. We have previously reported on the association of road traffic noise and air pollution finding the impact of individual exposure assessment compared to joint exposure can exaggerate risk [26]. In this work we model data on noise at different frequency components and air pollution using a QGIS software through machine learning to probe real-life problems of road traffic noise and air pollution related to hypertension cases and rate. This study used data on registered hypertension cases and their rate as measures of hypertension health outcomes. Geospatial modelling focuses on the analytical procedure used with a geographic information system (GIS) to characterise fundamental workings and characteristics for a specific collection of spatial features [25]. We formulated spatial maps to provide layer descriptions of the problem using the OSM model, and undertook spatial analysis of data, and interpretation of the model. The ultimate aim is to assess whether the approach can address the association between combined noise pollution and air pollution with hypertension cases and rate.

## 2. Materials and Methods

### 2.1. Study Design and Targeted Population

The study design is an analytical cross-sectional design. Road traffic air concentration and traffic exposure (noise at different components of frequencies) of community households in association with the prevalence of hypertension were assessed. This study was conducted to allow for data collection at one-time point.

The registered population with NHS Scotland is 5,626,383 million. Information from the NHS Scotland database of 776,579 hypertension patients’ registrations and the rates per 13.80 people were linked. The Scottish Health Board mid-year population estimate in Greater Glasgow households for private families is 1,152,172 million.

### 2.2. Data Source and Outcome

This work utilised an external database based on patients’ hypertension prevalence cases from the Information Services Division (ISD) 2020. The database includes all hypertension registers and hypertension rates covered by hospitals nationwide as part of the disease prevalence monitoring at GP offices in Scotland.

The data on hypertension prevalence were accessed by looking at GP patients’ records in Scotland [27]. The data consist of 17 illnesses, disease registrations, and rates per 100 people at the Scottish NHS Board, HSCP, Cluster, and GP practice levels (Full patient populations at the HSCP, NHS Board, and Scotland levels are available on the ISD website: (accessed on 13 December 2020) https://www.isdscotland.org/Health-Topics/General-Practice/Workforce-and-Practice-Populations/). Since the data are not accessible for all practices, illness registrations aggregated at the HSCP (and occasionally at the Cluster level) and higher levels are likely to be understated.

The following year after the fiscal year, the data are still relevant based on the statistics on disease prevalence. Total patient populations at the HSCP, NHS Board, and Scotland levels are provided on the (ISD) website [28].

The data source for this study includes 911 GP practices for the years 2018–19. Only GP practices open as of October 2019 are included in the data. Some GP practices did not upload data for several practices because the Quality and Outcomes Framework QOF Calculator no longer supported it after the QOF was terminated in 2016 [29]. Thus, the data are no longer accessible using the QOF calculator as of April 2019. The data can now be obtained through SPIRE [27].

The ISD is advancing SPIRE, a division of the NHS National Services Scotland (NSS), and the ISD and several health-related duties are merging into a new national public organisation named Public Health Scotland (PHS) [28]. Any references to the NSS in these resources are no longer valid because PHS has taken over the management and delivery of the SPIRE project from the NSS [28].

This study was conducted along the roads in residential areas of Greater Glasgow and Clyde. Five monitoring sites were chosen to gather the statistics (Glasgow High Street, Glasgow Towhead, East Dunbartonshire Bishopbriggs, East Dunbartonshire Kirkintilloch, and Glasgow Kerbside) due to their location in Scotland’s central region, which is marked by dense urban residential areas and busy significant roadways.

These sites were used because they have significant traffic flows with traffic noise and are in close proximity, which aids in the easy transportation of equipment, monitoring campaigns and other field study materials.

The prevalence of hypertension among different categories of private households in Scotland was investigated. In different localities, the exposure related to population density and nearest neighbours were considered.

The monitoring sites were set up and included five major urban traffic roads within in the city centres around 500 residential areas. Based on their similar locations at postcodes, these places were extrapolated from the Scottish Air Quality website, considering major-traffic road sites with heavy traffic noise and air pollution. The estimated background air pollution maps (2018) [30] at the total annual mean concentrations, following the grid squares of 1 km × 1 km, were retrieved [30]. Numerous coordinates are included in this DEFRA data set for every region of Scotland. For this modelling, the NO_2_**,** PM_10_, and PM_2.5_ yearly daily average air concentrations from five locations in Scotland’s Greater Glasgow from 23 November 2020, to 24 November 2021, were used. The Openair LAQM Data Download Site was used to collect the data [30].

### 2.3. Data Exclusion

Each patient population with hypertension registrations and the rates per 100 people shown at the Scottish NHS Board, HSCP, Cluster, and GP practice levels were geocoded, so no patient was disqualified for a missing geocode.

### 2.4. Environmental Data Collection and Modelling Approaches

The first phase of this study underwent noise monitoring to assess community households’ environmental emission and road traffic exposure (noise levels at different frequencies).

Five monitoring locations were chosen to set up the instruments at an area within the centre of Glasgow, which is characterised by heavy traffic on major roads. A Casella CEL-63x & 1/3 Octave Band Sound Level Meter was placed during the field monitoring at 1.2–1.5 m on a tripod and at 1 m from the roadside next to the air quality testing apparatus. In Glasgow, the sensors were frequently at least 10 metres from the centre line of the closest traffic lane (depending on the road type). It was consistently and safely set back from the road and at least one metre from any structure to prevent reflections from different site locations, as described previously in detail [26].

The concentrations of chemical pollutants were monitored from road traffic using Scotland’s air quality network data on NO_2_**,** PM_10_, and PM_2.5_ and were recorded as hourly averages.

The measurement was computed into annual average exposures to noise (Lday (daytime), Leve (evening time), and Lnight (nighttime)) for the five locations. Noise was modelled considering the (latitude x and longitude y) centroids in Greater Glasgow using the QGIS software and opeNoise plugins.

The QGIS offers many plugins. One is the opeNoise, which allows users to compute the noise level produced by a point source or a road source at fixed receiver spots and structures and has been described previously in detail [26].

Another processing tool used was the inverse-distance weighting (IDW). The IDW was used to generate air quality interpolated in a location enhanced by a point source or a road source to form raster layers and structures central to the receiver spots. This method has been described previously in detail [26].

The simulations were run for a variety of times, specifically for data obtained using the UK Calculation of Road Traffic Noise (CRTN) method and the CNOSSOS-EU standard method during the day at 14 h, in the evening at 2 h at a penalty of +5, and during the night at 8 h at a penalty of +10. [31,32]. When the data for the three reference periods (daytime, evening time, and nighttime) were set, the plugin automatically calculated the value of noise level at Lden. The Scottish air quality data on air pollution were exclusively gathered from urban metropolitan areas.

The hourly mean concentrations for nitrogen dioxide were used. For each pollutant, the boundaries were set between the index points.

The daily mean concentration for PM_2.5_ particles served as the basis for the current day’s most recent 24 h running mean. Based on the past PM_10_ particle data, the daily mean concentration generated the 24 h running mean for the present day.

### 2.5. Prevalence of Hypertension and Modelling Approaches

The prevalence of hypertension focuses on the patient register of reported hypertension cases and the rate of hypertension.

Data visualisation was enabled by opening the openaq.csv file with a text editor, and each row of data contains the information from a single monitoring station. The latitude and longitude columns include the hospital coordinates, while the value column has the average hypertension register cases and rate. When necessary for modification, the files were downloaded into Excel, modified by removing all disease prevalence except hypertension register and hypertension rate, and saved in the CSV format.

The delimited CSV format files are text files containing tabular data and may be imported into the QGIS using the Data Source Manager. By clicking the button, one may access the Data Source Manager.

The steps include the following: Navigate to the openaq.csv file and open it. Then, import this file as points, and choose the point coordinates—X Field for longitude and Y Field for latitude. Choose EPSG 4326—WGS 84 in the Geometry CRS. From the drop-down option, choose Add.

The tabular data are input in the QGIS canvas as a spatial data layer, where clicking on any of the points reveals the attribute information related to each issue. By opening the Layer Styling Panel (left click), selecting, and changing the single band grey to single band pseudocolour renderer from the drop-down menu. Choose and add a hospital symbol from the drop-down option.

To model the data into multiple shapefiles’ maps, spatial analysis on joint road traffic noise and air contaminants with hypertension was undertaken.

This kind of map comprises a vector layer and a raster layer with attribute information. Some attribute information is indicated in (Table 1). The geospatial analysis in geometric locations includes information on noise, air contaminants, residential structures, and roads. The report comprises data on individual characteristics (i.e., demographics, medical history, and lifestyle) and registered hypertension cases for each monitoring site.

### 2.6. Statistical Data Analysis

Statistical data analysis was performed using R analysis, Python Consoles, and QGIS 3.24.3-Tisler. Moran’s I was used to analyse the spatial autocorrelation of combined noise level and air pollutants (Lden, NO_2_**,** PM_10_, and PM_2.5_), using 100 simulations and a range of neighbours (nearest neighbourhoods) of 10 to 100 km and 1 km × 1 km grid squares in the QGIS. An analysis of spatial autocorrelation using Moran’s I was used to assess whether a registered hypertension case was an isolated case or was clustered, utilising several 100 simulations together with the number of neighbours (nearest) in a contiguous polygon.

A multivariate scatterplot relationship analysis was used to assess the mutual association between noise level, air concentration, hypertension rate, and registered cases. The distance correlation of vectors with values of the joint noise level at different frequency components and air concentration and hypertension rate were analysed using a proximity matrix (measure). The relationships between combined exposure to road noise at different components of frequencies, air pollution, and hypertension registered cases were examined using a multiple logistic regression.

Using the multiple logistic regression, we examined the relationships between combined exposure to road noise at different components of frequencies, air pollution, and hypertension rate. A 3D scatterplot relationship analysis was used to assess the mutual association between noise levels at different frequency components, air concentration, and hypertension rate.

The python plugin called Plotly in the QGIS enables the development of 3D clustering charts (multivariate scatterplots) with the help of the Plotly library and the Python API. The plots are completely interactive [33]. The algorithm used to create the clusters can be seen as being graphically correlated with the features and identified. However, there are spatial outliers and regional multivariate clusters [34]. Therefore, a multivariate scatter matrix analysis was used to examine if one significant cluster is visible.

### 2.7. Canonical Correlation Analysis

The relationships between the combined exposure to road noise and air pollution, hypertension registers, and hypertension rate were examined using non-linear canonical correlation (NLCC)

The steps for the NLCC approach are as follows: Finding one or more non-linear canonical functions, also referred to as canonical variables, that describe the various data dimensions is the first stage in the NLCC approach [35]. The highest correlation that could be determined from the sets of qualitative variables is represented by the first canonical variable. The ideal scaling level for the qualitative variables under consideration in the analysis determines the maximum number of canonical variables. The matching eigenvalue for each canonical variable is determined.

The percentage of the total variance explained by a specific canonical variable is represented by an eigenvalue, which is an index of variance in the range of 0 to 1. The fit value and the loss value are evaluated to assess how well the NLCC solution matches the qualitative data in terms of the relationship between the sets. The loss value indicates the discrepancy between the maximum fit value (the number of canonical variables) and the fit value, which represents the sum of the eigenvalues. In real life, percentages (0% to 100%) are used to express each of these values [35].

Finding the most pertinent variables regarding each canonical variable comes next after obtaining the variables. To perform this step, the NLCC approach calculates the canonical loading, also known as the structural correlation coefficient, for each research variable. A canonical loading, like principal component and factor analysis, shows the relationship between the input variable and the output canonical variable [35].

Like the Pearson’s correlation coefficient, this is interpreted. Any research variable with a canonical loading of 0.30 or higher is chosen as a significant variable for inferring the exposure–outcome relationship [35].

A canonical loading of less than 0.30 shows that a variable has little to no effect on the canonical variable.

The direction of the association is shown by the positive or negative sign of the canonical loading, much like in a traditional correlation study.

### 2.8. Two Sets of Categorical Variables

Set-1. Pollution: nitrogen dioxide (NO_2_), fine particulate matter (PM_2.5_), coarse particulate matter (PM_10_), high-noise-frequency component, low-noise-frequency component, and mid-noise-frequency component.

Set-2. Hypertension: hypertension registers and hypertension rate

## 3. Results

### 3.1. Geospatial Results of Data

**Figure 1:**
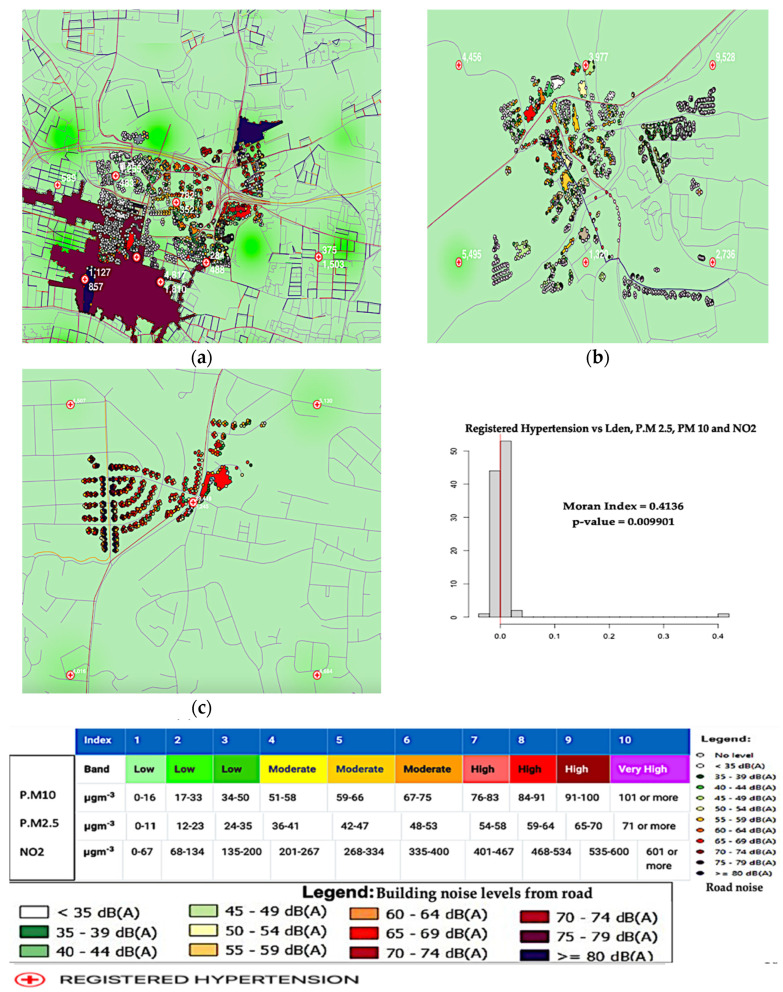
Joint annual average NO_2_**,** PM_10_, and PM_2.5_ concentrations (μg/m^3^) and noise level (Lden) at five monitoring locations (23 November 2021): (**a**) joint air–noise pollution and hypertension at GK, GT, and GHS; (**b**) joint air–noise pollution and hypertension at EDK; and (**c**) joint air–noise pollution and hypertension at EDB.

The NO_2_**,** PM_10_, and PM_2.5_ concentration(μg/m^3^) and noise level in Lden, calculated from daytime, nighttime, and evening noise level dB(A), are all included in the data. In this study, the data on hypertension prevalence were used for “Data Visualization”, as displayed below in Figure 1.

For hypertension registrations, the Scottish NHS Board, HSCP, Cluster, and GP practice levels are presented in Figure 1. Hypertension registrations aggregated at the HSCP level (and occasionally at the Cluster level) were not used since these data are not accessible for all practices. This result description contains the 2019 hypertension prevalence at the GP offices based on their identification number, the total population of hypertension register, and the rate.

We examined the joint annual average of air pollutants (μg/m^3^), noise pollution at Lden, and hypertension register.

The model is statistically significant, as shown in Figure 1a–c, because its *p*-values are below the 5% significance level. A value of 0.009901 is the *p*-value. The results suggest that hypertension is a clustered condition rather than an isolated one, meaning there is clustering on the map. The assumption that the observations are independent of one another is not supported if there is clustering in a map. The red vertical line on the plot represents the distribution of the Moran’s I values and the value of 0.4136 observed.

The MC simulation gave a *p*-value of 0.01, representing a 1% risk of mistakenly rejecting the null hypothesis or a 1% possibility that our observed pattern is consistent with a random process. The Moran’s spatial autocorrelation is positive. Therefore, the value of 0.4136 is closest to one.

Our findings depict that hypertension is significantly influenced by NO_2_**,** PM_10_, PM_2.5_, and Lden at all monitoring locations. The positive association between pollution and hypertension increases with increasing pollution by NO_2_**,** PM_10_, PM_2.5_, and Lden.

### 3.2. Multivariate Analysis and Scatter Matrix Analysis of Joint Air Concentration and Noise Level at Frequency Component Range with Hypertension Rate and Registered Hypertension Cases

**Figure 2:**
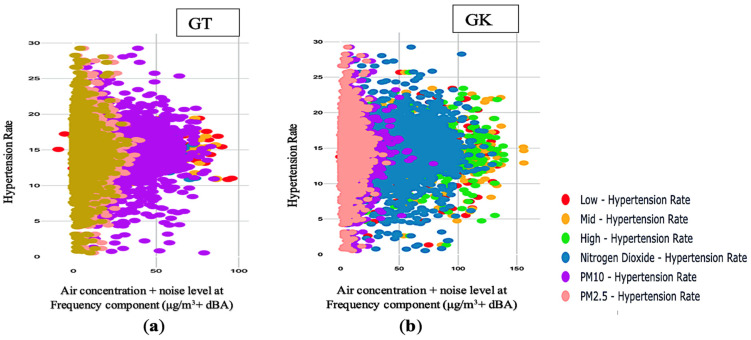

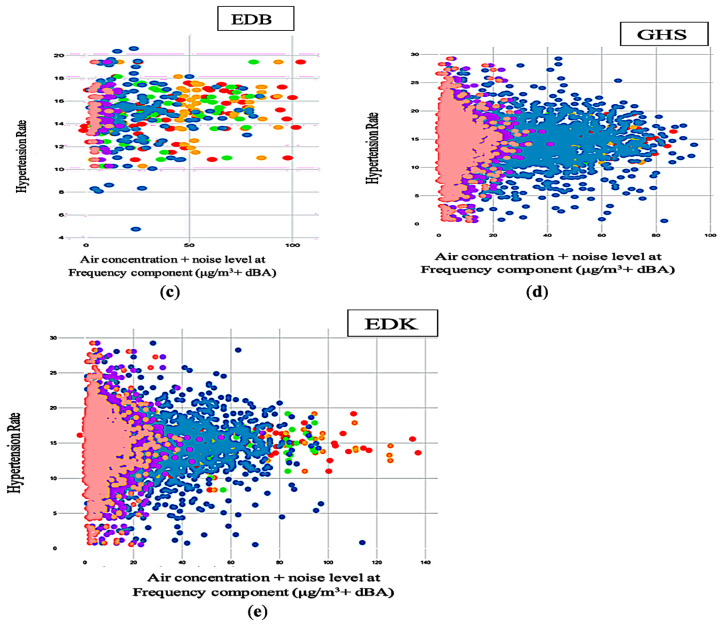
Relationship between joint air concentration and noise level at different frequency component range and hypertension rate at the five monitoring sites: (**a**) Joint air-noise pollution (dBA)/(μg/m^3^) with hypertension rate at GT; (**b**) Joint air-noise pollution (dBA)/(μg/m^3^) with hypertension rate at GK; (**c**) Joint air-noise pollution (dBA)/(μg/m^3^) with hypertension rate at EDB; (**d**) Joint air- noise pollution (dBA)/(μg/m^3^) with hypertension rate at GHS; (**e**) Joint air-noise pollution (Lden)/(μg/m^3^) hypertension rate at EDK.

Figure 2a–e shows a positive relationship between joint air concentration and noise level at different frequency component range and hypertension rate. As shown in Figure 2a–e, it moves in the same direction at all monitoring sites. Figure 2a–e seems to be a bell curve showing a Pearson correlation and a normal distribution at all the monitoring sites, except for EDB in Figure 2c. Figure 2a–e also shows a general nonlinear correlation at all monitoring areas, except for EDB in Figure 2c. From Figure 2a–e there is a clustering of data across all the monitoring sites, except for EDB in Figure 2c. On the other hand, as shown in Figure 2a–e, there are also outliers at all monitoring sites. The strength of the correlation is strong because there is clustering across all monitoring areas. This means hypertension is not an isolated case but a clustered one. This indicates that there is clustering on the map. Therefore, a mutual association exists between joint air concentration and noise level at the frequency component range and hypertension rate.

**Figure 3:**
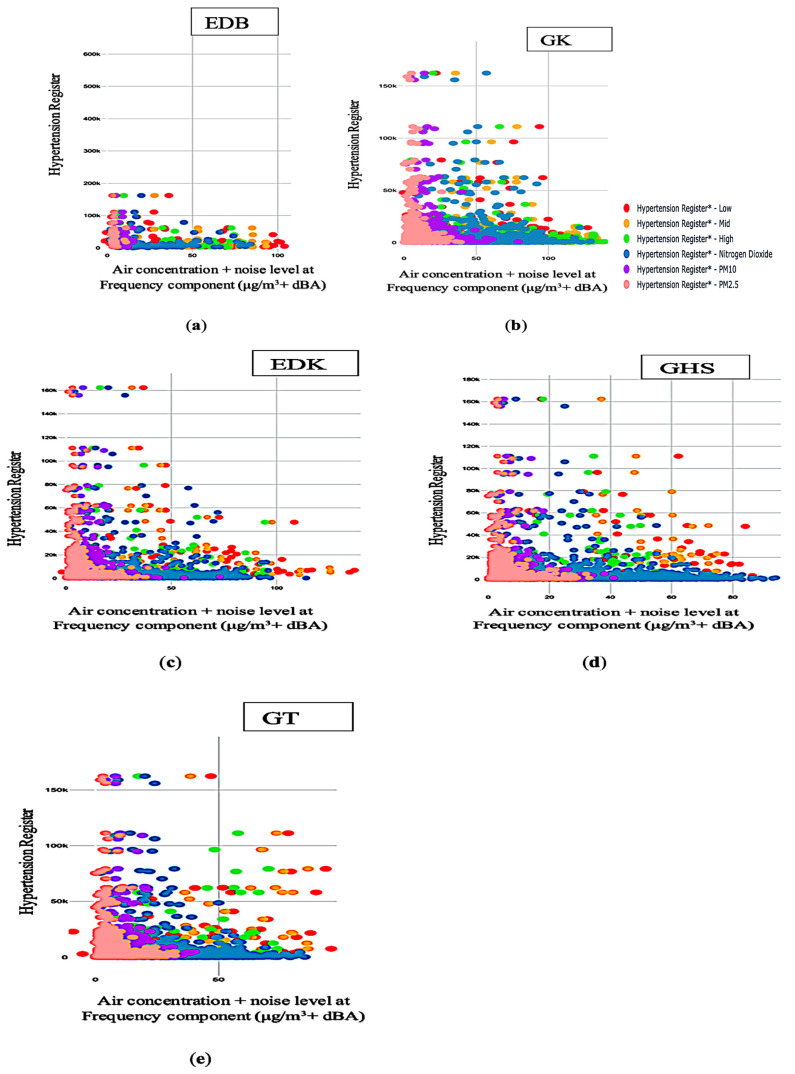
Relationship between joint air concentration and noise level at different frequency component range and registered hypertension cases at the five monitoring sites: (**a**) Joint air-noise pollution (dBA)/(μg/m^3^) with registered hypertension cases at EDB; (**b**) Joint air-noise pollution (dBA)/(μg/m^3^) with registered hypertension cases at GK; (**c**) Joint air-noise pollution (dBA)/(μg/m^3^) with registered hypertension cases at EDK; (**d**) Joint air- noise pollution (dBA)/(μg/m^3^) with registered hypertension cases at GHS; (**e**) Joint air-noise pollution (Lden)/(μg/m^3^) with registered hypertension cases at GT.

Figure 3a–e depicts a positive association between air concentration, noise level at different frequency components, and registered hypertension cases. Figure 3a–e shows this relationship moves in the same direction at all monitoring sites. From Figure 3a–e, there seems to be moderate clustering at all monitoring sites. Figure 3a–e also depicts a linear correlation at all monitoring areas, except for EDB in Figure 3a. As shown in Figure 3b–e, there are outliers at all monitoring sites. Figure 3a–e shows some outliers also exhibit clustering features at all monitoring sites. The strength of the correlation is moderate because there is moderate clustering across all monitoring areas. This means the registered hypertension cases are not isolated cases but clustered ones. This suggests a unique collection of registered hypertension cases reported to GP practices that are clustered together in time and geography. This indicates that there is clustering on the map. Therefore, a moderate relationship exists between joint air concentration and noise level at the frequency component range and registered hypertension cases.

### 3.3. Multiple Regression Analysis of Joint Air Concentration and Noise Level at Frequency Component Range with Registered Hypertension Cases

Table 2 shows the impact of noise level at low-, mid-, and high-frequency components and air concentrations of NO_2_**,** PM_10_, and PM_2.5_ μg/m^3^ on registered hypertension cases. The R² value of 0.14 reveals that the predictors explain 14% of the variance in the outcome variable, with F (1, 238) = 11.63, 10.57, 6.51, 14.70, and 12.89 and *p* < 0.001, 0.001, 0.011, <0.001, and <0.001, respectively. The findings reveal that NO_2_**,** PM_10_, PM_2.5_, mid-, and high-frequency component positively and negatively predict registered hypertension cases (β = 0.24, −0.40, 0.30, 0.75, and −0.72 and *p* < 0.001, 0.001, 0.011, <0.001, and <0.001, respectively). However, noise level at the low-frequency component has a non-significant effect on registered hypertension cases (β = −0.17 > 0.05). NO_2_ is likely to increase the risk of hypertension.

Table 3 shows the impact of noise level at low-, mid-, and high-frequency components and air concentrations of NO_2_, PM_10_, and PM_2.5_ (μg/m^3^) on the hypertension rate. The R^2^ value of 0.09 reveals that the predictors explain 9% of the variance in the outcome variable, with F (6, 190) = 3.17, *p* < 0.05. The findings reveal that PM_2.5_ and noise at the mid-frequency component negatively predict the hypertension rate, with (β = −0.29, *p* < 0.05) and (β = −0.66, *p* < 0.05). The findings also reveal that noise at the high-frequency component and PM_10_ positively predict the hypertension rate, with (β = 0.61, *p* < 0.05) and (β = −0.37, *p* < 0.05). In contrast, noise level at the low-frequency component and NO_2_ have a non-significant effect on the hypertension rate (β = −0.17 > 0.05).

The 3D scatterplot shows the impact of joint air concentration and noise at different frequency components (low-, mid-, and high-frequency components) on pollution and the hypertension rate identified in the areas. Noise level at different frequency component results in hypertension. However, pollution from road traffic noise at different frequencies combined with air concentration also increases the hypertension rate.

**Figure 4:**
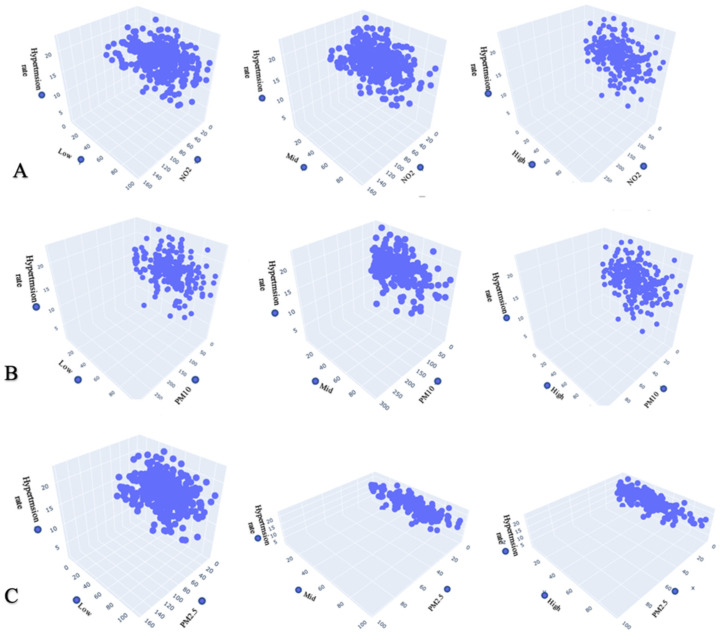
A 3D scatterplot showing the correlation between air concentration and noise level at different frequency components and hypertension rate.

Figure 4A shows a positive relationship between joint PM_2.5_ and mid-frequency component and hypertension rate. The standard noise level at the mid-frequency component and PM_2.5_ increase when the hypertension rate also increases. These findings mean that when joint PM_2.5_ and mid-frequency component rises for a year, the rate of hypertension will also increase. These findings show that an annual increase in daily pollution from joint PM_2.5_ and mid-frequency components from road traffic will cause the hypertension rate to increase annually.

Figure 4B shows a general positive linear correlation between joint noise level at the high-frequency component and PM_10_ with hypertension. These findings mean that the standard noise level at the high-frequency component and PM_10_ increase when the hypertension rate increases.

Figure 4C depicts a general negative linear correlation of joint noise level at the mid-frequency component and PM_2.5_ with hypertension rate. These findings connote that the hypertension rate also decreases when the communal noise level at the mid–frequency component and PM_2.5_ decrease. The correlation strength is strong across all the scatterplots, except for the joint noise level at the low-frequency component and NO_2_, which has a weak correlation with the hypertension rate. Across all the scatterplots, there seems to be a strong relationship between joint noise level at the high-frequency component and PM_10_ and hypertension rate. Therefore, a strong association between PM_2.5_ and noise level at the mid-frequency, combined with noise level at the high-frequency component and PM_10_, predicts the outcome of hypertension rate. The similarities across all the scatterplots show that the data groups are very close, indicating a correlation between the vector values across all the scatterplots.

### 3.4. Proximity Measure for Attributes between Vectors of Values

Table 4 shows that the correlation between distance using the proximity measure and nearest neighbours shows clustering, illustrating a correlation between the vector values and similarities. There is a similarity matrix between noise level at different frequency components, air concentration, and hypertension rate. This means a stronger correlation between noise level at different frequency components, air concentration, and hypertension rate.

The canonical correlation analysis explored the correlation between combined noise at different frequency components and air concentration and combined registered hypertension cases and hypertension rate.

**Figure 5:**
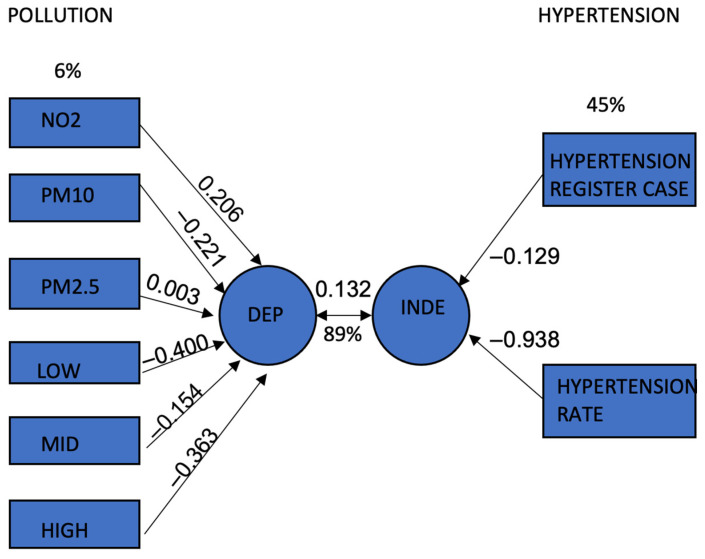
Canonical correlation between joint noise at different frequency components combined with air concentration, registered hypertension cases, and hypertension rate.

Figure 5 and Table 5 shows that the canonical correlation is 0.342. For the independent canonical variables, the canonical loading of NO_2_, PM_10_, PM_2.5_, low-, mid-, and high-frequency component is 0.206, −0.221, 0.003, −0.400, −0.154, and −0.363, respectively. The total variance of the six independent variables is 6%. The canonical loading of registered hypertension cases and hypertension rate is −0.129 and −0.938, respectively. The total variance of the two dependent variables is 45%. These findings mean that when PM_10_ and noise at the low-, mid-, and high-frequency components decrease, the registered hypertension cases and hypertension rate will also reduce. In addition, as NO_2_ and PM_2.5_ increase, the registered hypertension registered cases and hypertension rate will also increase.

From Figure 5 and Table 6 the variance explained by the canonical independent variable at a canonical correlation of 0.342 is 89%. Therefore, the correlation is significant at *p* < 0.05. This indicates significant correlations between joint air concentration and noise at different frequency components, registered hypertension cases, and hypertension rate.

## 4. Discussion

This study shows the impact of the joint relationship between air concentration and noise level at different frequency components on hypertension rate and registered cases.

The noise readings in Greater Glasgow show significant noise levels across the neighbourhoods monitored in this study. Practically every measuring point shows that the noise levels are higher than the values established for the designated study areas of research, which include the city centre; workshop, commercial, and administrative areas with housing; and zones along highways, main roads, and city traffic arteries; the noise levels are 65 dB(A) for day/evening. At the measuring point of the specified locations of GHS, EDK, and GK in the afternoon, the equivalent noise level reaches values of 86.8 dB(A) and 87 dB(A), respectively.

The results of the field noise measurements and a study of the traffic regime, which examined the volume of traffic at the recorded locations, were used to build a noise pollution simulation.

The air quality index shows low for one, two, and three (band). At all five monitoring sites, the yearly average NO_2_, PM_10_, and PM_2.5_ concentrations for air pollution levels ranged from 0–35,0–50, and 0–200 (μg/m^3^, respectively). Recommendations and health guidance for these low bands advise that people in those locations can engage in their typical outdoor activities without increased risk from air pollution. This implies that all building occupants are subjected to the same maximum levels of traffic noise and air pollution, which is not usually the case; thus, this type of simulation is not always acceptable. The noise level and air concentration data were evaluated in several different places at specified points in this study to confirm that the simulation was valid.

The spatial associations were studied between the modelled traffic-related noise, air pollution levels, and prevalence of hypertension. Both noise and air pollution showed different spatial patterns, and the correlations across the monitoring areas varied substantially, depending on the unit of analysis: Greater Glasgow, nearest neighbourhoods, and 1 km × 1 km grid squares. The auto spatial correlations across all monitoring areas in Greater Glasgow were slightly strong. They varied depending on the exposure metrics between NO_2_**,** PM_10_, and PM_2.5_, and the noise level (Lden) at a Moran’s I value of 0.413 is closest to one.

The auto spatial relationships were largely constant throughout the deprivation terrains, exposure terrains, and distance to significant road crossings. The red vertical line on the plot, which represents the distribution of Moran’s I values, represents the observed 0.4136 Moran’s I value. The MC simulation’s *p*-value of 0.01 indicates a 1% possibility that the null hypothesis has been mistakenly rejected or that our observed pattern is compatible with a random process. The Moran’s I value of 0.4136 is near one. Hence, it shows a positive spatial autocorrelation.

This indicates that hypertension is being influenced significantly by NO_2_**,** PM_10_, PM_2.5_, and noise level (Lden) at all monitoring locations. It means that hypertension increases with increasing pollution by NO_2_**,** PM_10_, PM_2.5_, and noise level (Lden).

A related study with somewhat different results [22] shows how to use a spatial unit and local features to analyse the association between air pollution and road traffic noise in London between 2003 and 2010.

Road traffic causes high noise levels that exceed the safe limits advised by the OSHA, the WHO, the Environmental Protection (Act) of 1990, the Noise and Statutory Nuisance Act of 1993, and local noise regulating organisations, according to the noise monitoring results in the chosen communities. Road traffic noise exposure in several localities is over the safe limits. Despite the low air pollution levels, some levels exceed the acceptable amount [36,37]. The annual average PM_2.5_ concentrations should not exceed 5 g/m^3^, and the 24 h average exposures should not exceed 15 g/m^3^ and more than 3–4 days per year, according to the new guidelines.

It is appropriate to use the WHO and EU air quality criteria while considering the present policy and air quality index [38,39].

All the types of pollution considered have the potential to harm people’s overall health, quality of life, and social communication. It is essential to avoid any public health impacts. It is appropriate for local and national government to consider implementation of noise reduction measures in the assessed monitoring sites and areas.

The study findings show a positive relationship between joint PM_2.5_ and noise level at the mid-frequency component and hypertension rate. As the joint noise level at the mid-frequency component and PM_2.5_ increase, the hypertension rate also increases and moves in the same direction. Our findings depict that the rate of hypertension will also increase when joint PM_2.5_ and noise level at the mid-frequency component rises for a year. This shows that an annual increase in daily pollution exposure to joint PM_2.5_ and noise level at the mid-frequency component from road traffic will cause the hypertension rate to increase annually.

The findings show a general positive linear correlation of joint noise level at the high-frequency component and PM_10_ with hypertension rate. When the hypertension rate increases, the joint noise level at the high-frequency component and PM_10_ increases, and vice versa. The findings also depict a general negative linear correlation of joint noise level at the mid-frequency component and PM_2.5_ with hypertension rate. This means that when the joint noise level at the mid-frequency component and PM_2.5_ decreases, the hypertension rate also decreases. There is a strong correlation across all the scatterplots, except for joint noise level at the low-frequency component, and NO_2_ correlates with the hypertension rate. Therefore, a strong association of PM_2.5_ and noise level at the mid-frequency, combined with noise level at the high-frequency component and PM_10_, predicts the outcome of hypertension rate. A comparable study was previously carried out [40]. A higher incidence of hypertension was linked to increased ozone levels in a large cohort of African American women. Although the incidence of hypertension decreased with higher NO_2_ levels, higher NO_2_ levels were not linked to increased rates of hypertension [40].

Furthermore, the correlation of distance using the proximity measure and nearest neighbours shows clustering, illustrating a correlation between the vector values and similarities. The similarities across all the scatterplots show that the data groups are very close, indicating a correlation between the vector values across all the scatterplots.

A conventional way to present statistical visualisation is the 3D scatterplot. It uses a cloud of points to represent the joint distribution of two variables, with each point denoting a dataset observation. From this representation, one may deduce a lot about whether there is a significant relationship between them.

The 3D scatter plot in this study shows the relationship between joint air concentration and noise at different frequency components and the hypertension rate identified in the areas. The noise levels at different frequency components result in hypertension. However, pollution from road traffic noise at different frequency combined with air concentration also increases hypertension rate.

A scatter matrix is made up of numerous pairwise scatterplots displayed as a matrix. The matrix reveals a positive correlation between joint air concentration and noise level at different frequency components and hypertension variables. It assists us in estimating whether there is a correlation between various factors. It allows us to plot a selected scope over two metrics with their corresponding aggregation.

Scatterplots can only show two factors, although data are frequently clustered using numerous variables. Multiple ways have been considered to visualise more than three variables, but each has drawbacks.

The size of the markers is used to create a bubble chart, but that is not the best option. Since the variables would have different encodings, the third variable could not be compared with the others. For instance, noise level or air concentration has a higher or no effect by glancing at the chart previously created. In this study, hypertension rate or registered hypertension cases as a third variable were included and compared to the other two.

In addition to encoding the third variable, 3D charts can be confusing and occasionally even deceptive. Nevertheless, 3D scatterplots can be helpful, particularly if they are dynamic. Current technologies can fully support the analysis and visualisation of differential expressions. Furthermore, 3D plots are typically non-static and permit interaction to comprehend the many components of the data using different parameters, which increases the effectiveness of data exploration. Therefore, a multivariate scatter matrix analysis was used to examine if one significant cluster was visible. Here, there was a lot of clustering effect on all the monitoring sites, except for the EDB monitoring site, showing that the hypertension rate is dispersed and not clustered cases.

Our work is the first to explore an association of joint air concentration and frequency components of road traffic noise with the prevalence and rate of hypertension. The findings revealed that NO_2_**,** PM_10_, PM_2.5_, mid-, and high-noise frequency positively and negatively predicted registered hypertension cases (β = 0.24, −0.40, 0.30, 0.75, and −0.72, *p* < 0.001, 0.001, 0.011, < 0.001, < 0.001), whereas low-frequency component had a non-significant effect on registered hypertension cases (β = −0.17 > 0.05). The findings revealed that PM_2.5_ and noise at the mid-frequency component negatively predicted hypertension rate, with (β = −0.29, *p* < 0.05) and (β = −0.66, *p* < 0.05).

In addition, noise at the high-frequency component and PM_10_ positively predicted registered hypertension cases (β = 0.61, *p* < 0.05) and (β = −0.37, *p* < 0.05). In contrast, noise level at the low-frequency component and NO_2_ had a non-significant effect on (β = −0.17 > 0.05). This means that when PM_10,_ noise at low-, mid-, and high-frequency components decreases, hypertension cases and rates will also reduce. In addition, it depicts that as NO_2_ and PM_2.5_ increase, registered hypertension cases and hypertension rate will also increase. Similar research revealed statistically significant differences in NO_2_ [13]. It showed heart failure association by gender (men had the largest association), baseline hypertension (hypertensive individuals had the strongest association), and diabetes (strongest association among diabetics). Similar patterns could be detected for noise, but the interactions lacked statistical significance [13].

The variance explained by the canonical independent variable at the dependent canonical correlation of 0.342 is 89%. Therefore, the correlation is significant because *p* < 0.05. Hence, there is a significant correlation between joint air concentration and noise at different frequency components and combined registered hypertension cases and hypertension. The biggest canonical loading value among the pollution variables in the first canonical variable is −0.400, which indicates “noise at low frequency components”. This finding supports the hypothesis that low-frequency (10–200 Hz) traffic noise exposure may cause hypertension by irritating the cortical or subcortical regions via the neuroendocrine system [12]. High-frequency component of traffic noise is the second pollution variable with a high canonical loading value. Hypertension rate and cases show the highest canonical loading values for the second canonical variable. It is a common and important health issue in Europe and UK [1,2,3,4]. In the UK, data from a health survey estimating the prevalence of hypertension around areas in England revealed that about 11.8 million adults had hypertension, equating to 22.6% of the population [2]. The Scottish Public Health Observation [3] estimates that the risk of hypertension is high and can lead to significant death. The prevalence sharply increased among people aged 16 years and above in 2019 [3]. Hypertension in Wales increased by 15.8% between 2018 and 2019 and is currently the most prevalent morbidity [4]. Nonetheless, according to the Northern Ireland Quality and Outcome Framework Statistics (QOF), the prevalence rate of hypertension is 14.0%, making it the most prevalent morbidity in Northern Ireland [5]. The joint air concentration and noise at different frequency components are correlated with registered hypertension cases and hypertension rate, as demonstrated by the second canonical variable.

The “pollution” (set 1) and “hypertension” (set 2) categorical variables, as represented by the NLCC technique, are linked. As seen in Figure 5, the graphical representation of all correlations (canonical loadings) aids researchers in understanding the similarities and differences between the study variables as well as the multidimensional patterns in the data, especially for those who are unfamiliar with multivariate techniques.

As additional indicators of hypertension, GP’s patient reports on the prevalence of hypertension were used to empirically determine the registered patient population and hypertension rate conditions from the registry. Numerous personal social, economic, and lifestyle characteristics could be considered.

Due to the strong association, it is constrained to combine the effects of noise exposure and air pollution exposure in this investigation.

Current research that classifies subjects based on the findings of environmental measures has grown [41]. Additionally, it is more efficient to track different frequency elements of air concentration and road traffic noise to analyse the link between these features and the prevalence of hypertension.

Even though this strategy has been used in numerous studies [42,43,44], a registered case of reported history of hypertension that has been medically diagnosed has a sensitivity range of 33.3 per cent to 71 per cent and a specificity of 91 per cent to 96 per cent [42,45]. However, the sensitivity might change depending on the age group and population.

That study did not account for socioeconomic position as we did in our analysis. Our study considered that those who had lived within 100 m of the five main roadways shared an equal socioeconomic standing.

As a result, measuring road traffic noise during weekdays at daytime may understate their noise levels, thus leading to exposure misclassification bias in our study.

In this study, fewer changes in the statistical analyses were made by using raw data, and the relationships might not simply be chance findings. They represent accurate correlations since the results are consistent with the hypothesis that low–high frequency noise-induced discomfort contributes to the development of hypertension [11,12].

This study was unable to interview people to collect data on the prevalence of hypertension and measure blood pressure due to the uncertainty of the COVID-19 pandemic and the response of the ethical committee. The committee could not permit face-to-face interviews due to the COVID-19 regulation. However, secondary data could be used [42,46]. Approval was obtained from the University of West of Scotland’s health and safety department to conduct fieldwork safely during the COVID-19 pandemic. To be safe to conduct the research, the air quality monitor station provided by the government was set up on the roadside to conduct road noise assessment. This approach catered to future uncertainty and enabled the research to be conducted, regardless of the COVID-19 restrictions during the pandemic.

This study examined multiple relationships between registered hypertension cases and hypertension rate and exposure to road traffic noise at various frequency components and air concentration. This investigation had no contrasting vulnerabilities (all came from the same source). Future research should be conducted using optimal samples. The noise measures only considered a person’s immediate exposure to road traffic noise and their long-term exposure. A 1 h LAeq measuring was employed at the five chosen sampling sites to evaluate the relationship between road traffic noise frequency components and hypertension. Prior research has shown that short-term noise levels may be a reliable indicator of long-term noise levels [47].

## 5. Conclusions

This research extends our previous work to demonstrate the associations between traffic noise, air quality, hypertension rate, and registered hypertension cases. It identified the urban areas where environmental noise levels and air concentration are high enough to disrupt several aspects of residents’ life in the studied city.

This type of research serves as a starting point and a foundation for further developing noise pollution and air pollutant mapping with hypertension across the entire Greater Glasgow, as well as strategic maps, to contribute to disease modelling in the field of epidemiology studies.

Creating a strategic map in Glasgow would make noise and air quality management much easier locally. Strategic maps, for example, offer information on the state of the environment. Current and previous data on air pollutants and noise pollution can be used in the background to predict the risk of hypertension and the rate of hypertension cases.

Noise and air concentration can be estimated by visualising places where the prescribed limit values have been exceeded on the strategic maps. In addition, the number of people and households are counted on the register of GPs or hospitals in specific areas exposed to noise and air pollutants above the limits for being at risk of hypertension. Building a strategic map on joint air concentration and noise pollution with hypertension will significantly aid and expedite future traffic development planning and public health in Greater Glasgow, which must be implemented.

Rapid urban development without accompanying growth in economic opportunities has led to a greater number of individuals in urban communities. Inadequate regulation of activities in this sector, noise exposure, and repeated exposure to high levels of noise from road traffic are likely to lead to a risk of hypertension among households. This study conducted at community sites within Greater Glasgow set out to determine the related risk of community indoor and outdoor noise exposure and air pollution on hypertension among the sampled sites using noise measurement, air concentration, and prevalence of hypertension based on registered cases and hypertension rate.

A GIS enabled assessment has a great capacity to integrate and model diverse data from different sources, including spatial and descriptive components, into one framework. It has the potential to become an effective evidence-based practice tool for identifying and resolving problems early in the community health process. When used correctly, GIS assessment can inform and educate (professionals and the public); empower decision-making at all levels; assist in planning and fine-tuning clinically and economically effective actions, assist in predicting outcomes before making any financial commitments and in assigning priorities in an environment of limited resources; change practices; and continuously monitor and analyse changes. Associating GIS managed datasets analyses and processing with data openness, such as openness with regard to data on air quality, medical informatics, and data integration, would eventually result in the promotion of sustainability and city growth through public involvement and sophisticated data processing.

These data can help investigate the impact of the joint association between road traffic, environmental noise, and air pollution on the prevalence of hypertension.

The modelling of noise and air pollutant data with hypertension in geospatial views can be used by researchers in different disciplines, such as public health, epidemiology, computing and engineering medical science, geography health science, and urban planning and ecosystem research.

It is concluded that exploring the joint association in the spatial correlation between traffic-related noise at different frequency components and air concentration exposure is essential to evaluate the impact of the potential combined association of noise and air pollution on hypertension registered cases and rate. It is important to carefully assess the spatial unit of a study, and if data are available, statistical models should consider the within-unit distribution of correlations.

## Figures and Tables

**Table 1:** Parameters for creating a 3D scatterplot for the vector layers.

Label	Name	Type	Description
Input layer	INPUT	[vector: any]	Input vector layer
X attribute (air pollution)	XFIELD	[table field: NO_2,_ PM_10,_ PM_2.5_ ]	Field to use for the *x*-axis: [NO_2,_ PM_10,_ PM_2.5_]
Y attribute (noise frequency)	YFIELD	[table field: LOW, MID, HIGH]	Field to use for the *y*-axis: [LOW, MID, HIGH]
Z attribute (hypertension rate)	ZFIELD	[table field: Hypertension rate]	Field to use for the z-axis: [Hypertension rate]
Histogram	OUTPUT	[html] Default: [Save to temporary file]	Specify the HTML file for the plot. Choose one of the options. Save to File
Histogram	OUTPUT	[html]	HTML file with the plot. Available in the Processing ► Result Viewer.

**Table 2:** The regression coefficients of joint noise level at low-, mid-, and high-frequency components and air concentrations of NO_2_, PM_10_, and PM_2.5_ (μg/m^3^) showing impact on registered hypertension cases.

Variables	B	SE	t	Sig.	95.0% CI
Constant	151.41	79.09	1.91	0.057	(−4.40, 307.22)
NO_2_	5.12	1.50	3.41	<0.001	(2.16, 8.08)
PM_10_	−28.86	8.88	−3.25	0.001	(−46.35, −11.37)
PM_2.5_	33.98	13.32	2.55	0.011	(7.75, 60.21)
LOW	−2.28	1.53	−1.49	0.137	(−5.29, 0.73)
MID	11.32	2.95	3.83	<0.001	(5.50, 17.14)
HIGH	−10.82	3.01	−3.59	<0.001	(−16.75, −4.88)

Note: CI = Confidence Interval.

**Table 3:** The regression coefficients of joint noise level at low-, mid-, and high-frequency components and air concentrations of NO_2_, PM_10_, and PM_2.5_ μg/m^3^ showing impact on hypertension rate.

Variables	B	SE	t	Sig.	95.0% CI
(Constant)	145.615	62.434	2.332	0.021	(22.46, 268.77)
PM_2.5_	−22.159	10.934	−2.027	0.044	(−43.73, −0.59)
HIGH	6.996	2.539	2.755	0.006	(1.99, 12.01)
NO_2_	−2.000	1.237	−1.617	0.107	(−4.44, 0.44)
PM_10_	17.470	7.304	2.392	0.018	(3.06, 31.88)
LOW	1.655	1.233	1.343	0.181	(−0.78, 4.09)
MID	−7.225	2.405	−3.004	0.003	(−11.97, −2.48)

Note: CI = Confidence Interval.

**Table 4:** Distance correlation between the vector values of joint noise level at different frequency components and air concentration and hypertension rate using a similarity matrix.

SECTION A	NO_2_	LOW	Hypertension Rate
NO_2_	1.000	−0.131	0.122
LOW	−0.131	1.000	0.012
Hypertension Rate	0.122	0.012	1.000
	NO_2_	Hypertension Rate	MID
NO_2_	1.000	0.122	−0.082
Hypertension Rate	0.122	1.000	−0.016
MID	−0.082	−0.016	1.000
	NO_2_	Hypertension Rate	HIGH
NO_2_	1.000	0.122	−0.107
Hypertension Rate	0.122	1.000	0.033
HIGH	−0.107	0.033	1.000
^1^ Similarity matrix SECTION B	Hypertension Rate	PM_10_	LOW
Hypertension Rate	1.000	0.053	0.129
PM_10_	0.053	1.000	0.014
LOW	0.129	0.014	1.000
	Hypertension Rate	PM_10_	MID
Hypertension Rate	1.000	0.053	0.041
PM_10_	0.053	1.000	0.023
MID	0.041	0.023	1.000
	Hypertension Rate	PM_10_	HIGH
Hypertension Rate	1.000	0.053	0.110
PM_10_	0.053	1.000	0.001
HIGH	0.110	0.001	1.000
^1^ Similarity matrix SECTION C	Hypertension Rate	PM_2.5_	LOW
Hypertension Rate	1.000	−0.007	0.129
PM_2.5_	−0.007	1.000	0.118
LOW	0.129	0.118	1.000
	Hypertension Rate	PM_2.5_	MID
Hypertension Rate	1.000	−0.007	0.041
PM_2.5_	−0.007	1.000	0.159
MID	0.041	0.159	1.000
	Hypertension Rate	PM_2.5_	HIGH
Hypertension Rate	1.000	−0.007	0.110
PM_2.5_	−0.007	1.000	0.133
HIGH	0.110	0.133	1.000

^1^ Similarity matrix.

**Table 5:** Canonical correlation and Eigen values.

	Correlation	Eigenvalue	Wilks Statistic	F	Num D.F	Denom D.F.	Sig.
1	0.342	0.132	0.869	2.160	12.000	356.000	0.013
2	0.127	0.016	0.984	0.590	5.000	179.000	0.707

**Table 6:** Variance and Eigen values.

	Eigen Value	Variance %
1	0.132	89
2	0.016	11
Total	0.148	100

## Data Availability

Not applicable.
